# Gestational weight gain and its determinants among pregnant women in Gurage zone, Central Ethiopia: a cohort study

**DOI:** 10.1186/s12905-024-03223-8

**Published:** 2024-06-28

**Authors:** Girma Alemayehu Beyene, Mukrem Abdulwehab Yunus, Aberash Beyene Deribew, Abebaw Wasie Kasahun

**Affiliations:** 1 Department of Public Health, College of Medicine and Health Sciences, Wolkite University, Wolkite, Ethiopia; 2 Department of Internal Medicine, College of Medicine and Health Sciences, Wolkite University, Wolkite, Ethiopia; 3 Department of Midwifery, College of Medicine and Health Sciences, Wolkite University, Wolkite, Ethiopia

**Keywords:** Gestational weight gain, Pregnancy outcome, Birth-weight

## Abstract

**Background:**

The nutritional status of the mothers before pregnancy and the weights gained during pregnancy are very crucial factors affecting the pregnancy outcomes and health of the infants. This study aimed to assess early pregnancy weight, determine the magnitude of gestational weight gain, and investigate the factors affecting gestational weight gain among pregnant women in the Gurage zone, 2022.

**Methods:**

A prospective cohort study was conducted among pregnant women who started antenatal care follow-up before the 16th week of gestation in the selected hospitals and health centers of the Gurage zone, Ethiopia. The gestational weight gain was obtained by subtracting the early pregnancy weight from the last pregnancy weight and categorizing based on the Institute of Medicine (IOM) recommendation.

**Results:**

The early pregnancy weight status of the women at enrollment indicates that 10% of them were underweight and 83% of them had normal weight. On average, the study participants gained 13.3 kgs of weight with [95% CI: 13.0, 13.6]. More than half (56%) of them gained adequate weight, a quarter (26%) of them gained inadequate weight, and 18% of them gained excess weight during pregnancy compared to the IOM recommendation. Maternal age, occupational status, and early pregnancy weight status were found to have a statistically significant association with the gestational weight gained.

**Conclusion:**

Almost half (44%) of the pregnant women gained either inadequate or excess weight during pregnancy. Promoting gestational weight gain within recommended guidelines should be emphasized for younger, employed women and those who are either underweight or overweight.

## Background

Even though, a global rise in obesity affects people of all ages, women of reproductive age are at a high and growing risk. In Africa and Ethiopia, more women are becoming overweight before and during pregnancy. In Southern Ethiopia, over a quarter of adults have too much weight and women are more than twice as likely to be overweight as men [1–5].

The amount of weight gain during pregnancy can have implications for the health status of both the mother and the infant in the short and long term. Excessive gestational weight gain may lead to complications such as high blood pressure, gestational diabetes, and changes in biochemical and hormonal levels that affect fetal growth and development. These complications can increase the risk of cardiovascular disease later in life. Therefore, maternal weight gain during pregnancy affects not only obstetric outcomes but also health outcomes in middle and old age [6–10].

To reduce obstetric risks and have a favorable pregnancy outcome the Institute of Medicine (IOM) in collaboration with the research council of the National Academies published the revised recommended gestational weight gain based on the World Health Organization (WHO) Body Mass Index (BMI) categorization as indicated in below (Table 1) [11].

**Table 1:** Institute of Medicine Recommended gestational weight gain based on the early pregnancy BMI of the women

Early pregnancy BMI of the women	Recommended gestational weight gain
< 18.5 kg/m^2^	12.5–18 kgs
18.5–24.9 kg/m^2^	11.5–16 kgs
25-29.9 kg/m^2^	7-11.5 kgs
≥ 30 kg/m^2^	5–9 kgs

A study conducted in Mexico and Iran revealed half of the pregnant women gained weight higher than the IOM recommendation. Maternal age, antenatal follow-up, family size, food insecurity, stress, anxiety, and violence were factors identified to be associated with weight gain during pregnancy [12, 13]. A study in Malaysia, Malawi, Brazil, and Ghana showed roughly two-thirds of the pregnant women gained gestational weight more than the IOM recommendation. Marital status, employment status, early pregnancy nutritional status, and dietary patterns were significantly associated with gestational weight gain [14–20].

In the Tigray region of Northern Ethiopia, the average gestational weight gain was 10.6kg. Almost two-thirds of pregnant women did not meet the IOM recommendation of gestational weight gain [21]. A study conducted in the Harari region of Eastern Ethiopia indicated the mean gestational weight gain was 8.96 kgs and, only 28% of the pregnant women gained adequate weight as per the IOM recommendation. Early pregnancy nutritional status and antenatal care follow-up were found to have a statistically significant association with gestational weight gain [22].

Evidence is scarce in the study setting and this study aimed to evaluate early pregnancy weight, measure the amount of weight gain during pregnancy, and identify the factors that influence weight gain among pregnant women in the Gurage zone, 2022. The findings of this study will be used by the decision-makers and concerned stakeholders who aim to improve maternal and child health by preventing obstetric complications. Moreover, this study is intended to be used as a baseline for further study on the effects of gestational weight gain on pregnancy outcomes.

## Methods

### Study area

The study was conducted in the Gurage zone, which is one of the zones in the southern nations and nationalities' regional states in Ethiopia. The capital of the zone is Wolkite town which is located 158 km southwest of the national capital, Addis Ababa. The zone has 13 woredas and 5 town administrations. According to the Ethiopian Statistical Services, the zone's projected total population for 2023 is 1,870,368. This includes 963,197 females and 907,171 males [23]. According to the data obtained from the Zonal Health Department, there are nine functional hospitals in the zone, seven of which are public, and two of them are owned by non-governmental organizations. In addition, there are 64 governmental and six non-governmental Health centers and 412 Health posts in the zone.

### Study design

This study followed a cohort of pregnant women who attended antenatal care services in hospitals and selected health centers in the Gurage zone. The study included pregnant women who started antenatal care before the 16th week of gestation. The study participants were recruited from April 18, 2022, and were followed until March 09, 2023.

### Study population

The study population for the research consisted of a cohort of pregnant women who attended prenatal care visits at selected health facilities in the Gurage zone before the 16th week of their pregnancy.

### Inclusion criteria

Pregnant women with singleton pregnancies who came for antenatal care visits before the 16th week of gestation and with no known comorbidities like Diabetes Mellitus (DM) and hypertension were included in the study.

### Sample size determination

The sample size required for the study was calculated using Epi Info ™ 7 software using a 95% confidence level, 90% power, 24.23% of the normal weight, and 41.12% of the under/overweight women gained adequate gestational weight from a previous related study [22], which gives a sample size of 344. Adding 25% to account for the loss to follow-up, the final sample size targeted for this study was 430.

### Sampling technique and procedures

Three hospitals and five health centers were selected through a process of simple random sampling technique facilitated by Microsoft Excel Professional Plus 2021. This method involved the use of the “=RANDBETWEEN” function to generate random numbers, which were assigned to each potential health institution within the study setting. Subsequently, those institutions corresponding to the randomly selected numbers were chosen to participate in the study. Considering the limited percentage of pregnant women with early antenatal care follow-up in their early pregnancy and the low prevalence of institutional delivery, consecutive sampling of pregnant women meeting the inclusion criteria was applied until the required sample size was achieved.

### Data collection techniques and procedures

Data were collected by trained and experienced data collectors using structured and pretested questionnaires. The questionnaire was designed after related journal articles [12, 22, 24] were reviewed and translated into the local language of Amharic and administered with the Amharic version.

The questionnaire included questions about the participants' sociodemographic background, as well as their reproductive and obstetric history. The weight scale was calibrated and verified with known weights to ensure its accuracy, and the women were asked to wear light clothing and remove their shoes. The data collector measured the weight of the women in kilograms while they stood at the center of the scale, and recorded it on the questionnaire.

The woman's height was measured using a stadiometer with the woman standing straight. She was instructed to remove any shoes or accessories that could affect her height and to keep her hair flat and not styled in a way that could alter the measurement. The data collector read the measurement from the stadiometer and recorded it in centimeters on the questionnaire. The measurement was rounded to the nearest 0.1 centimeter. Then, the Body Mass Index (BMI) was determined by dividing the women’s weight (in kilograms) by the square of their height (in meters).

All the completed questionnaires were checked for consistency of the results and very meticulous supervision was made daily. Data documentation codebooks were used to make data entry forms in Epi Info software and check commands were applied to restrict inappropriate and inconsistent values.

### Variables

#### Dependent variables

- Gestational weight gain

#### Independent variables

- ➢ Sociodemographic and Obstetric factors
  - Age, marital status, religion, educational status, occupation,income, parity, height, weight, gestational age
- ➢ Early pregnancy weight status: Underweight, Normal weight, Overweight

### Operational definitions and definitions of terms

- **Early pregnancy weight**: weight measured before 16 weeks of gestation at the time they were enrolled in the study [9].
- **Last pregnancy weight**: The final pre-delivery weight measured just before delivery [25].
- **Gestational weight gain**: calculated as the difference between the last pregnancy weight measured just before delivery and early pregnancy weight measured before the 16th week of gestation, and categorized according to the 2009 IOM gestational weight gain recommendations (inadequate, adequate, or excess) [9, 21, 26, 27].

### Data processing and analysis

Data were entered into Epi Info 7 and exported to STATA version 17 for cleaning and analysis. The background characteristics of the study participants were summarized by the descriptive statistics and results were presented in narration, tables, and figures as deemed appropriate.

The distribution of gestational weight gain among different maternal early pregnancy weight status and age categories was assessed graphically using a box and whisker plot. The mean gestational weight gain for each category of the maternal weight status was calculated and the Analysis of Variance (ANOVA) test was used to check the statistically significant difference in mean gestational weight gained between the mothers of different weight status and age categories. Bartlett's equal-variances test was used to test the assumption of homogeneous variances. The corresponding significance level of more than 0.05 was used for failing to reject the homogenous variance assumption. Multiple comparison test was performed using the Bonferroni test and the results were presented as a matrix. The mean difference between each of the categories was calculated and Bonferroni adjusted significance of the difference was used to decide between which group a significant difference was observed.

We also used linear regression analysis to examine how gestational weight gain related to other factors. The multiple linear regression model fitness was assessed using the F-statistics test and its p-value of less than 0.05 indicated that there is at least one independent variable linearly related to the gestational weight gained. Variance inflation factor (VIF) was calculated to check the existence of potential multicollinearity of all the independent variables. Since the maximum and mean values of the calculated VIF for all the independent variables were 2.60 and 1.63 respectively, there was no multicollinearity problem.

## Results

### Sociodemographic characteristics

Out of the 430 pregnant women who were invited to join the cohort, six of them had missing data on some key variables in their questionnaires. Therefore, 424 respondents' data were used for the final analysis. The age of the study participants ranges from 16–40 years with a mean ± standard deviation of 26.5 ± 6.4. Two in five of the study participants were less than 25 years of age. Most of the study participants were married (87.26%), more than a third (37.74%) of the study participants were orthodox, 70.76% of them completed secondary school education or more, and more than half (51.89%) of them were employees. The monthly income of the household ranges from 1000–15000 Ethiopian Birr (ETB) with a mean ± standard deviation of 6160.1 ± 2619.2 (Table 2).

**Table 2:** Sociodemographic characteristics of the study participants in Gurage zone, 2022

Variables	Categories	Frequency	Percent
Age (Mean ± SD = 26.52 ± 6.37) Median = 27 years	15–19	80	18.87
	20–24	90	21.23
	25–34	215	50.71
	35–40	39	9.20
Marital status	Single	38	8.96
	Married	370	87.26
	Divorced	9	2.12
	Widowed	7	1.65
Religion	Orthodox	160	37.74
	Muslim	53	12.50
	Catholic	89	20.99
	Protestant	101	23.82
	Others^a^	21	4.95
Educational status	No Formal education	15	3.54
	Primary school	109	25.71
	Secondary school	152	35.85
	College and above	148	34.91
Occupation	Housewife	143	33.73
	Employee	220	51.89
	Student	61	14.39
Income in Ethiopian Birr (Mean ± SD = 6160.1 ± 2619.2)	< 5000	142	33.49
	5000–10000	258	60.85
	> 10000	24	5.66

Others^a^: Include Jehovah’s Witnesses and Traditional Waaqefannaa religion

### Reproductive and obstetrics characteristics

**Fig. 1:**
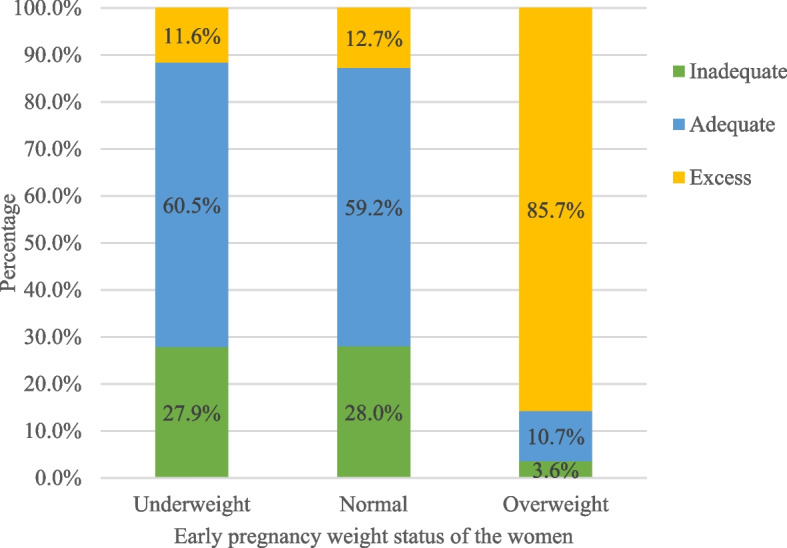
Gestational weight gain based on the early pregnancy weight status of the women, 2022

**Fig. 2:**
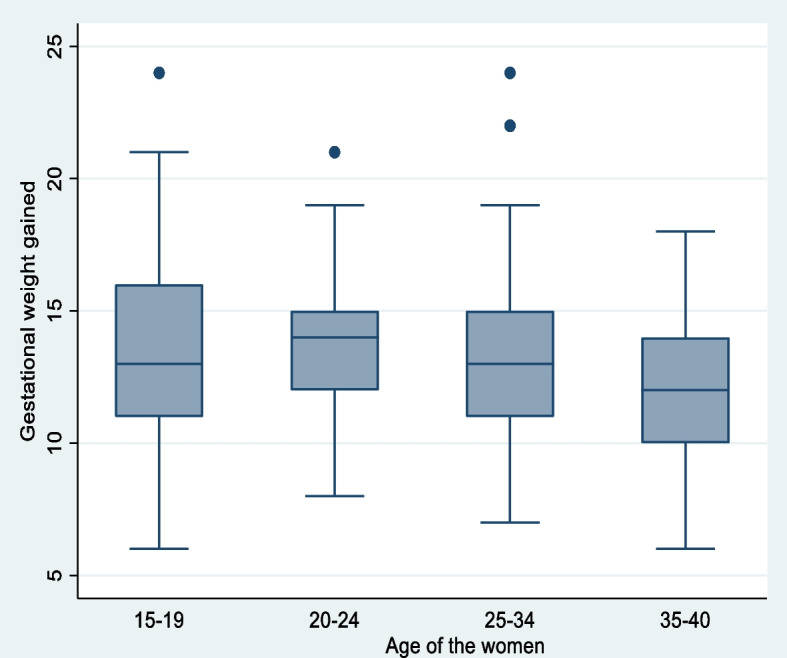
Gestational weight gain among different age categories of women who gave birth in the selected health facilities of Gurage zone, 2022

**Fig. 3:**
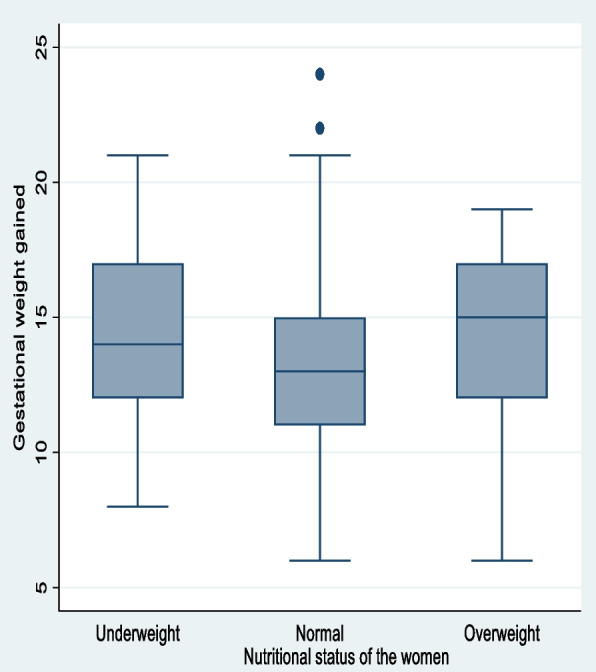
Gestational weight gain among the different early pregnancy weight status of women who gave birth in the selected health facilities of Gurage zone, 2022

Pregnant women in their early pregnancies ranging from 12-16th weeks were enrolled in the cohort study, with a mean gestational age of 13.9 weeks. The average number of gravidities per woman in the study was almost three, with a range of one to six pregnancies. The early pregnancy weight status of the women at enrollment indicates that 10% of them were underweight, 83% of them had normal weight, and 7% were overweight. The mean ± standard deviation of the weight of the children born to mothers in the study was 2791.4 ± 308.6 grams. On average the study participants gained 13.3 kgs of weight with [95% CI: 13.0, 13.6]. A quarter (26.42%) [95% CI: 22.4, 30.8] of the pregnant women gained inadequate weight, 17.45% [95% CI: 14.1, 21.4] of them gained excess weight and more than half (56.13%) [95% CI: 51.4, 60.8] of them gained adequate weight during pregnancy compared to the IOM recommendation. Furthermore, the percentage of inadequate, adequate, and excess weight gained by the study participants based on their early pregnancy weight status was presented by the stacked bar graph (Fig. 1). The gestational age at which the study participants gave birth ranged from 34–42 weeks with a mean of 38 weeks. The distribution of gestational weight gained among different age categories and maternal early pregnancy weight status was almost normal (Figs. 2 and 3).

### Factors affecting gestational weight gain

A one-way ANOVA test indicated that there is a statistically significant difference in the mean gestational weight gained by women with different age categories; F (3, 420) = 2.76, *p* = 0.0418. Women in the age group of the early twenties gained more weight, 13.75 kg on average (SD = 2.57) compared to women in their late thirties who gained 12.15 kg average weight (SD = 3.22) (Table 3).

**Table 3:** ANOVA test displaying the difference in mean gestational weight gain based on maternal age group, 2022

Summary					
Maternal Age group	Mean gestational weight gained			Std. dev.	Freq.
15-19	13.43			3.16	80
20-24	13.76			2.57	90
25-34	13.39			2.93	215
35-40	12.15			3.22	39
Total	13.36			2.95	424
Analysis of variance					
Source	SS	Df	MS	F	Prob > F
Between groups	71.30	3	23.77	2.76	**0.0418**
Within groups	3614.21	420	8.60		
Total	3685.51	423	8.71		
Comparison of Gestational weight gained by Age (Bonferroni)					
Row Mean- Col Mean	15-19			20-24	25-34
20-24	0.330				
	1.000				
25-34	-0.039			-0.369	
	1.000			1.000	
35-40	-1.271			-1.602	-1.232
	0.162			**0.028**	0.097

Bartlett's equal-variances test: chi2(3) =   4.382    Prob>chi2 = 0.223

Statistically significant at *p*<0.05

A statistically significant difference in the mean gestational weight gained was observed based on maternal early pregnancy weight status, as revealed by the ANOVA result; F (2, 421) = 6.37, *p* = 0.0019. Examining the F-ratio, we see that the value is 6.37 with a significant level of 0.0019 which is less than 0.05. Based on the results, we can reject the null hypothesis that the mean gestational weight gain is the same across different categories of maternal weight status. The data shows that women who were underweight or overweight before pregnancy gained more weight than those who had normal weight (Table 4).

**Table 4:** ANOVA test displaying the difference in mean gestational weight gain based on maternal weight status, 2022

Summary					
Early pregnancy weight status of the mothers	Mean Gestational weight gained			Std. dev.	Freq.
Under Weight	14.37			2.97	43
Normal	13.13			2.89	353
Overweight	14.64			3.04	28
Total	13.36			2.95	424
Analysis of variance					
Source	SS	Df	MS	F	Prob > F
Between groups	108.29	2	54.15	6.37	**0.0019**
Within groups	3577.22	421	8.49		
Total	3685.51	423	8.71		
Comparison of Gestational weight gain by Early pregnancy weight status of the mothers (Bonferroni)					
Row Mean-Col Mean	Underweight			Normal	
Normal	-1.239				
	**0.026**				
Overweight	0.271			1.509	
	1.000			**0.026**	

Statistically significant at *p*<0.05

To validate these results, multiple linear regression analysis was conducted and the result revealed a statistically significant association between maternal age, occupational status, and early pregnancy weight status, with the mean gestational weight gained. The model was also significant with F (20, 403) = 1.76, Prob > *F* = 0.0229. Marital status, educational status, religion, income, parity, and gestational age at delivery do not have a statistically significant association with the mean gestational weight gain (Table 5).

**Table 5:** Factors affecting gestational weight gain among women who gave birth in selected health facilities of Gurage zone, 2022

Gestational Wt. Gained	Coefficient	Std. error.	T	*P* > t	[95% conf. interval]	
Age category						
15–19	-0.36	0.50	-0.73	0.467	-1.35	0.63
20–24	Reference group					
25–34	-0.75	0.44	-1.69	0.088	-1.61	0.11
35–40	-1.89	0.68	-2.76	**0.006**	-3.24	-0.55
Marital status						
Single	-0.29	0.55	-0.53	0.595	-1.37	0.78
Married	Reference group					
Divorced	-1.39	1.02	-1.35	0.179	-3.37	0.63
Widowed	-0.43	1.13	-0.38	0.706	-2.65	1.78
Religion						
Orthodox	0.16	0.47	0.34	0.732	-0.77	1.08
Muslim	Reference group					
Catholic	0.15	0.52	0.29	0.770	-0.87	1.17
Protestant	0.18	0.49	0.36	0.716	-0.80	1.16
Others	0.78	0.77	1.01	0.311	-0.73	2.29
Educational status						
No Formal Education	-0.50	0.87	-0.58	0.562	-2.22	1.21
Primary school	0.49	0.39	1.25	0.213	-0.28	1.27
Secondary school	-0.02	0.36	-0.04	0.964	-0.73	0.69
College or above	Reference group					
Occupational status						
Employee	0.72	0.35	2.06	**0.041**	0.03	1.40
Student	0.35	0.53	0.65	0.517	-0.70	1.39
Housewife	Reference group					
Early pregnancy weight status						
Normal	Reference group					
Underweight	1.16	0.50	2.30	**0.022**	0.17	2.15
Overweight	1.66	0.59	2.82	**0.005**	0.50	2.83
Income	-0.00	0.00	-0.30	0.767	-0.00	0.00
Parity	0.20	0.12	1.73	0.084	-0.03	0.44
Gestational age at delivery						
Preterm	-0.18	0.42	-0.44	0.659	-1.00	0.64
_cons	12.83	0.69	18.41	0.000	11.46	14.20

Statistically significant at *p*<0.05

## Discussion

This study examined the weight status of pregnant women in their early pregnancy, the magnitude of gestational weight gain, and the factors affecting gestational weight gain among pregnant women in a low-income setting.

The findings of this study indicated, 10% of the pregnant women were underweight and seven percent of them were overweight. This value is much less than the study conducted in Addis Ababa in 2019 where two-thirds of the pregnant women were either overweight or obese. The proportion of underweight women was larger than in the study conducted in Iran, but those who were overweight were much smaller as compared to 39.6% of overweight women in Iran. A possible justification for the disparities observed in the early pregnancy status of pregnant women may include, socioeconomic and demographic determinants such as income level, and educational attainment. These factors may restrict access to nutritionally rich food sources, and awareness of the importance of nutrition during pregnancy, thereby impacting the dietary intake and, consequently, the weight status of these women. This assertion is supported by other articles and studies in the field [13, 24, 28–30].

The mean weight gain during pregnancy in this study was 13.3 kgs which is more than the study conducted in the Tigray region, Harari region, and Iran where 10.6 kgs, 8.96 kgs, and 11.3 kgs of average weight were gained respectively. This might be because most of the study participants were of normal weight which demands higher recommended weight gain. In our study, we did not have any participants who were classified as ‘obese’. However, in the studies we referenced, there were obese women for whom lower weight gain is recommended [13, 21, 22].

More than half (56%) of the study participants gained adequate weight in our study setting. This finding is more than the studies conducted in the Northern Ethiopian region of Tigray, Harari, Spain, and Thailand where one-third of the pregnant women gained adequate weight. Also, larger than the study conducted in Malawi and Malaysia where one in every five pregnant women had recommended gestational weight gain. This might be because of the different periods in which the studies were conducted and the difference in the measurement methods used as the study in Malawi measured weight in the second and third trimesters of pregnancy [17, 20–22, 31, 32].

The findings of the study indicated a statistically significant association between maternal age, occupational status, and early pregnancy weight status, with the mean gestational weight gained. Women in the age group of the early twenties gained more weight than women in their late thirties. This is in line with the study conducted in Harar and Iran where the nutritional status of the mothers and age were significantly associated with the weight gained during pregnancy. Mothers with older ages had reduced gestational weight gain [13, 17, 22]. However, the study conducted in Spain indicated no statistically significant association between maternal age and gestational weight gained [31].

One possible explanation for this finding is that younger mothers may have higher metabolic rates and energy needs than older mothers, which may result in increased food intake and weight gain. This hypothesis is supported by previous studies that have shown that maternal age is inversely related to basal metabolic rate and energy expenditure [33, 34].

The result of the study revealed a statistically significant association between maternal occupational status and the mean gestational weight gained. Specifically, employed women gained less weight during pregnancy compared to housewives. The possible justification for this includes that employed women may experience occupational demands, long working hours, and physical activity at work affecting energy expenditure, and potentially leading to lower weight gain.

The strength of the study lies in its identification of the determinants of gestational weight gain through a prospective follow-up study. However, it's indeed crucial to recognize the inherent limitations of our study. Under ideal circumstances, the calculation of gestational weight gain should be based on the difference between the weight just before delivery and the weight at conception. However, given the specific context of our research setting, preconception care is not a customary practice. To obtain the best estimates, we utilized the early pregnancy weight, specifically before the 16th week of gestation, as a surrogate for the weight at conception. This approach may lead to underestimating the actual gestational weight gain. However, it is important to note that the initial 16 weeks of pregnancy are generally characterized by minimal weight gain. Consequently, this factor is unlikely to significantly compromise our results' validity. Additionally, due to a limited percentage of pregnant women with early antenatal care follow-up, consecutive sampling of pregnant women meeting the inclusion criteria was applied to secure the required sample size. Since the presentations of the women to the antenatal care follow-up is at random this may not affect the validity of our results.

## Conclusion

The mean weight gain during pregnancy in this study was 13.3 kgs. Almost half (44%) of the pregnant women gained either inadequate or excess weight during pregnancy compared to the IOM recommendation. The result of this study revealed a statistically significant association between maternal age, occupational status, and early pregnancy weight status, with the mean gestational weight gained.

Promoting gestational weight gain within recommended guidelines should be emphasized in antenatal care to ensure better maternal and infant health. It is of paramount importance to provide antenatal counseling to expectant mothers regarding the maintenance of an optimal weight throughout their pregnancy. This information is particularly critical for younger, employed women and those who are either underweight or overweight. The health implications for both mother and child necessitate a heightened focus on this aspect of prenatal care.

## Data Availability

The datasets generated and/or analyzed during the current study are available from the corresponding author upon reasonable request.

## Abbreviations

ANOVA: Analysis of Variance
BMI: Body Mass Index
CI: Confidence Interval
DM: Diabetes Mellitus
GWG: Gestational Weight Wain
IOM: Institute of Medicine
SD: Standard Deviation
VIF: Variance Inflation Factor
WHO: World Health Organization
